# Health in global context; beyond the social determinants of health?

**DOI:** 10.3402/gha.v6i0.23506

**Published:** 2014-02-13

**Authors:** Anja Krumeich, Agnes Meershoek

**Affiliations:** Department of Health Ethics and Society, CAPHRI School for Public Health and Primary Care, Faculty of Health, Medicine and Life Sciences, Maastricht University, Maastricht, The Netherlands

**Keywords:** social determinants of health, health equity, health disparities, globalization, health promotion guidelines, evidence for practice, evidence for policy, local context, local adaptation

## Abstract

The rise of the social determinants of health (SDH) discourse on the basis of statistical evidence that correlates ill health to SDH and pictures causal pathways in comprehensive theoretical frameworks led to widespread awareness that health and health disparities are the outcome of complex pathways of interconnecting SDH. In this paper we explore whether and how SDH frameworks can be translated to effectively inform particular national health policies. To this end we identified major challenges for this translation followed by reflections on ways to overcome them. Most important challenges affecting adequate translation of these frameworks into concrete policy and intervention are 1) overcoming the inclination to conceptualize SDH as mere barriers to health behavior to be modified by lifestyle interventions by addressing them as structural factors instead; 2) obtaining sufficient in-depth insight in and evidence for the exact nature of the relationship between SDs and health; 3) to adequately translate the general determinants and pathways into explanations for ill health and limited access to health care in local settings; 4) to develop and implement policies and other interventions that are adjusted to those local circumstances. We conclude that to transform generic SDH models into useful policy tools and to prevent them to transform in SDH themselves, in depth understanding of the unique interplay between local, national and global SDH in a local setting, gathered by ethnographic research, is needed to be able to address structural SD in the local setting and decrease health inequity.

Since the end of the 1990s approaches to enhance the global population’s health are facing a revisiting of poverty and inequality discourses, with a central role ascribed to the so called Social Determinants of Health (SDH). Although the concept of SDH has been defined in many different ways across the literature and although it refers to a wide variety of factors and mechanisms, there seems to be consensus that while illnesses have biological causes, it is mainly social causes that explain the distribution of illness within a population or between populations (1). Social causes explain why some people have a higher risk of disease and of serious disability due to that disease than others. They also explain why the consequences of illness or disability have a more serious impact on the lives of the vulnerable than on those of the wealthy. SDH are the media through which the social world shapes a person’s health directly and indirectly and, as the literature on SDH shows, include an extensive and diverse range of variables such as poverty, social economic status, gender, age, ethnicity, educational level, access to adequate diet and safe water, access to adequate housing, living circumstances, habitual behavior, exposure to (occupational) hazards, exposure to pollution, waste management, exposure to violence, the ability to exert control over one’s own life and over circumstances linking to health, the class system, the housing stock, the educational system, the health care system, the labor market, public and social policy (1, 2), as well as the world market, and international trade and politics (3–8), the latter adding international dimensions to an individual’s chance of a healthy life, or more broadly, to (health) inequities across the globe.

At present the SDH discourse has entered many debates, discourses and theories. It has for example, been incorporated into current social psychological approaches in Health Promotion that traditionally focused primarily on individual characteristics and behavior. It also plays a central role in many policy and strategy discussions within the World Health Organization (WHO) and other international institutions.

In spite of health promotion’s traditional preoccupation with lifestyle and individual health behavior, recent Health Promotion models have adopted SDH as major constituents of the social, economic and political environment that impacts an individual’s lifestyle and health behavior options (9–12). As a consequence ‘ecological’ and ‘empowerment’ models are being introduced in Health Promotion that pay attention to power dynamics undermining a person’s chance to a healthy life, while empowering that person by giving that person tools (knowledge and awareness of health risks and of power issues) to control circumstances required to maintain a healthy lifestyle (10–12).

Moreover, since the 1990s the SDH debate has been picked up by the WHO which has since then initiated a series of policy and discussion papers focusing on the SDH as underlying causes of ill health, as well as of limited access to health care services (13, 14).

However, in spite of global consensus on the relevance of the SDH discourse for global health and on the need to integrate SDH and equity considerations into policy and program design, and in spite of its adoption by many fields and approaches little concrete progress seems to have been made (13, 15) and examples of effective, sustainable intervention and policy explicitly inspired by SDH frameworks are difficult to find. In this paper we will discuss some major models and theories on SDH and health equity. We will then reflect on potential challenges for translation of these frameworks into concrete policies and interventions that can explain the lack of progress in design and formulation of concrete, practical SDH based initiatives.

## Current models involving SDH

**Fig. 1:**
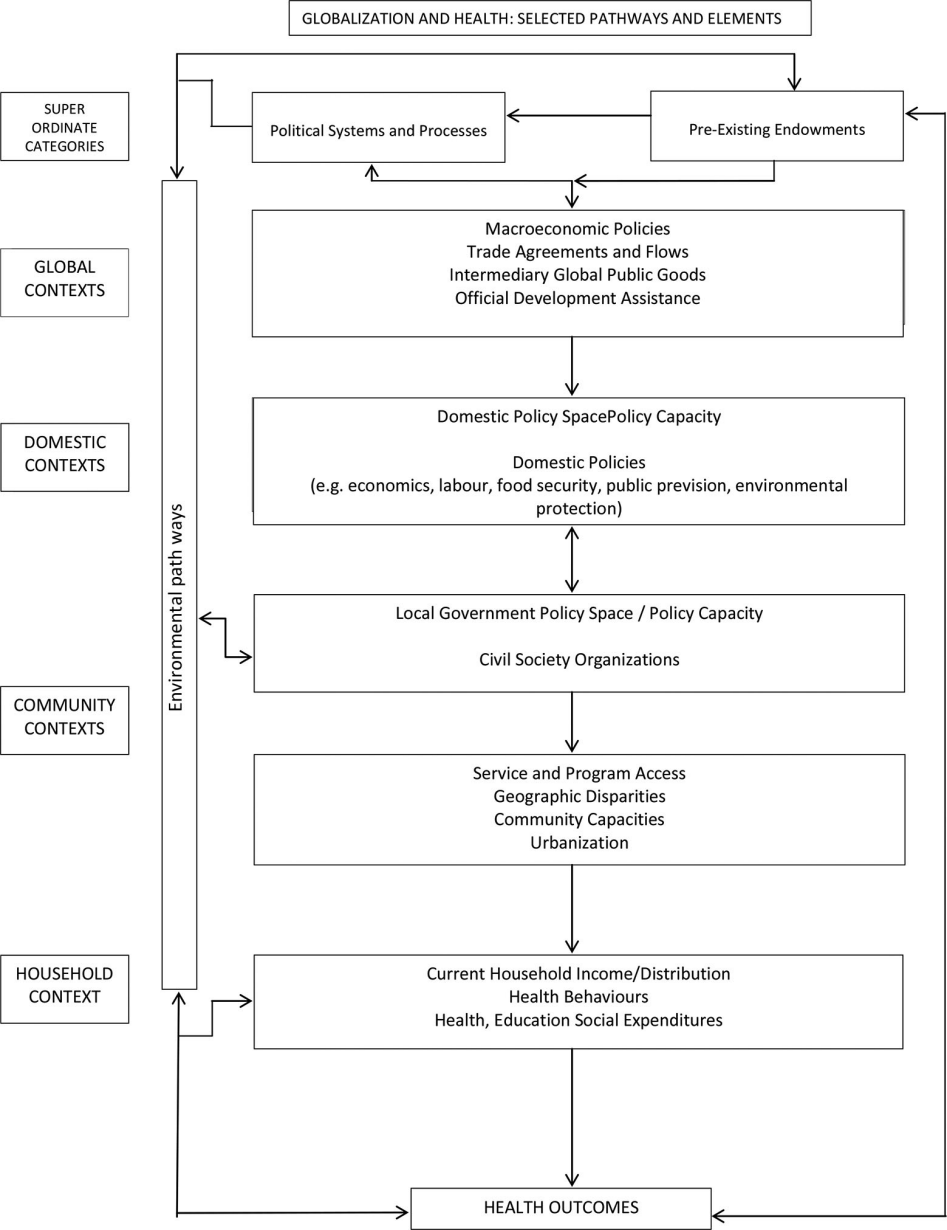
Globalization and health: basic framework. *Source*: Labonté and Torgerson (16).

Since the rise of the SDH discourse many models picturing the social production and reproduction of health have been constructed. These models differ with regard to focus, the determinants they include, as well as with regard to the levels of determinants they distinguish. An example of one of the more comprehensive models is the model on ‘globalization and health: selected pathways and elements’ by Labonté and Torgerson (16). They distinguish five levels of elements that function simultaneously to form complex pathways to health inequities (see Fig. 1).

Each element and each pathway can impact health outcomes directly as well as indirectly (i.e. through interconnecting with elements at the other levels). At the first level the ‘super ordinate level’ Labonté locates two major categories of elements: the political, economic and civil society traditions underlying society and the level of economic development, the availability of environmental resources and of human resources. The second level refers to the ‘global context’, to macroeconomic policies (such as structural adjustment programs and poverty relief strategies), trade agreements, global public goods, and development assistance and debt relief that determine the space for national governments to invest in regulation and public sector. The third level is the level of the ‘domestic context’ which determines local resources, endowments and opportunities as well as the space, openness and opportunities for civil society organizations and includes national labor policy, food security policy, migration and refugee policy, public provision policy, political power policy, environmental protection policy, and governmental macroeconomic policy. Level four, the level of ‘community contexts’, is the level at which access to programs and services is determined through geographic disparities, community capacity and urbanization. The level of ‘household contexts’ is the last level and includes household income and distribution, subsistence production, health behavior, and a household’s expenditures on health, education and other social services. Crosscutting the levels are what Labonté refers to as ‘environmental pathways’ that include international dimensions such as waste management, biodiversity, etc. Health outcomes in this model result from pathways connecting elements within and between the different levels.

**Fig. 2:**
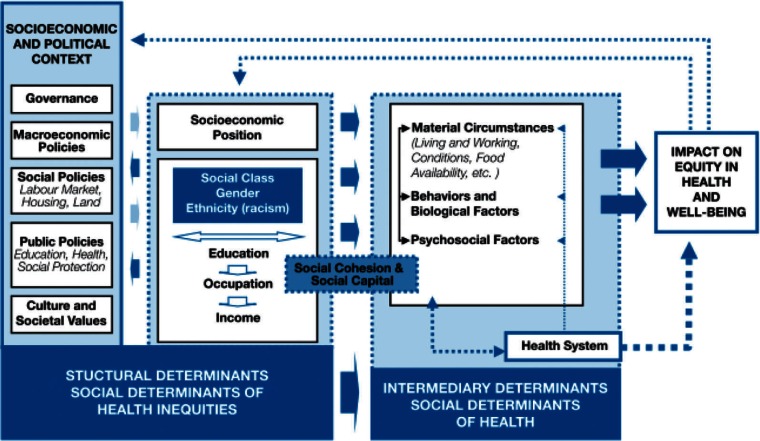
Final form of the CSDH conceptual framework. *Source*: WHO (1).

Another example is the framework by the WHO’s Commission on Social Determinants of Health (CSDH) (1). The framework provides an overview of major categories of determinants, but also pictures how these categories could interconnect (see Fig. 2).

Thus it aims to not only to depict the many potential impacts on health by SDH, but also to serve as a starting point and guide for policy, strategy and action. Starting from the assumption that health is defined by complexity, and that health is in essence a social phenomenon, the CSDH firmly grounds its framework in established theoretical traditions [material/structuralist theory; psycho-social model; social production of health model; eco-social theory (14)] and in a myriad of previous SDH models that view health and disease as products of social processes and that define the risk and the impact of ill health as the outcomes of inequalities in the distribution of SDH within or across populations. The framework distinguishes ‘structural determinants’ that include all social and political mechanisms (governance, macro-economic policy, social policy, public policy as well as social and cultural values) that generate, configure and maintain socioeconomic position (social class, gender or ethnicity) and ‘intermediary determinants’ that include not only working and living conditions, but also behavioral, psychosocial and biological factors and the health care system per se. Interactions between structural and intermediary SDH then determine differentiations (inequities) in health and well-being.

Neither Labonté’s model nor the CSDH framework is unique in their complexity. Similarly complex models, referring to many different levels of SDH from which health disparities result via direct or indirect pathways have been generated by Dahlgren and Whitehead (17), Barton and Grant (18), Huynen et al. (7), and many others. And although these models may vary at points [with Birn (6) for instance referring more explicitly to issues of power resulting from historical (colonial) relationships, and to social class, social position and social structure while including Social Determinants of Empowerment along with the SDH, and with others explicitly distinguishing between SDH per se and SDH inequities] all stress that health outcomes and or health inequities are shaped by multiple pathways that combine elements in many complex ways and that may function alone, simultaneously or in interaction with each other. What elements are included, what pathways they constitute, which pathways are involved, and how exactly they impact health inequalities, may differ from one location to another and in time. It has been pointed out not only, that if it comes to understanding local health and health disparities, everything can be related to everything in an endless number of ways, but also that analysis and understanding of the separate elements as well as of the dynamic and dynamically interacting pathways will need to be at the basis of any effective and sustainable policy, strategy and intervention (19).

Because of their awareness of the complexity of health, their careful identification and inclusion of all relevant SDH, their sound theoretical grounding, and their emphasis on the need for understanding these complexities for effective policy making and action, SDH models could inspire valuable tools for policy making in real life settings. International guidelines, tools and strategies for tackling inequities in the distribution of SDH, however, are largely unavailable (14) and many policy makers are struggling to anchor public and health policy and strategy in SDH frameworks.

Analysis of earlier attempts to base policy and intervention on insights in the way SDH impact local and global health reveals a number of potential challenges. Below we will discuss some of those challenges and reflect on ways to tackle them.

## Challenges to SDH approaches

Reviewing literature on challenges regarding translation of general SDH models into practical tools yields a large number of challenges. In this section we will discuss some of these challenges and their impact on health outcomes and health disparities.

### SDH and the neoliberal discourse

An important challenge is the continued dominance of a neoliberalist discourse tending to stress individual responsibility and resulting in a preference of lifestyle approaches focusing on individual behavior (20, 21). Deterred by the immense complexities involved in dealing with the social nature of health and by its association with its past political (i.e. socialist) agenda many policy makers and scholars prefer ‘individual’ models for their relative simplicity, small scale applicability and avoidance of an explicit socioeconomic and hence political agenda (4, 19). Although SDH are integrated in some of the life style models, they are often depicted as barriers to healthy behavior with health promotion activities focusingon empowering individuals to overcome these barriers. A classic example in this respect is that of women undergoing training to empower them by enhancing their negotiation skills (for safe sex), leading to increased violence against these women as they were violating traditional gender roles by claiming negotiation power and by showing knowledge of sexuality associated with commercial sex workers.

### Evidence for impact of SDH-interventions

A second challenge involves the construction of the evidence base on the SDH, in particular with regard to measurements involving the impact of action on the SDH. While the unequal distribution of social and economic determinants (income, employment, education, housing, physical and social environment) are known to produce inequalities in health (22), and while the general relationship between social factors and health is well established (2, 23), insight in the exact nature of this relationship is largely unavailable and, as a consequence, a basis for designing sustainable intervention is lacking (14).

Investigating the correlation between SDs and health, however is extremely difficult. As Aronowitz (24) demonstrates, the way disease states are framed and categorized in research and the way a cause or set of causes is attributed to those disease states, can be considered as a performative act and has the capacity to become self-perpetuating. The relationship between ethnicity and health, for instance, is often assumed or even established, showing that ethnic minorities have a lower health status than dominant ethnic groups, but without giving insight what exactly causes ill health in those ethic minority groups. What ethnicity entails is unclear and highly variable. In some studies ethnicity is linked with socio-economic status or education, and for instance limited access to material means that leads to limited access to proper food, housing etc. is seen as explanation for the correlation. In other studies ethnicity is understood as cultural background, which again can refer to a variety of different issues ranging from inadequate diet due to traditional notions of health and food, to wrong expectations concerning health professionals, divergent notions of body and health, cultural constructions of feminity and masculinity, cultural constructions of illness and risk etc. As Helberg-Proctor et al. (25) show in an analysis of Dutch studies on ethnicity and health, those more specific ‘determinants’ are quite often not measured in the studies; instead they are suggested potential explanations for the correlation between ethnicity and health that is established in the study.

Furthermore, Helberg-Proctor et al. show that the way ethnic categories are made is highly variable, implying that depending on the specific research project the same people can be categorized in different ethnic categories and thus supposed to have different characteristics. In other words, ethnicity is not so much a characteristic of people that can easily be determined (see also (26–28), but is constructed in research. Although the more specific aspects that are assumed to be responsible for the correlation between ethnicity and health are not measured as such, they are validated in research. This contributes to a process in which health inequalities become true, as they are the bases for training and education of professionals on how to deal with ethnic differences in health care practice. Meershoek et al. (29) for instance, show in a detailed analysis of the occupational health setting how assumptions concerning ethnic differences lead to differences in treatment and actually to a higher risk of permanent work disability. Paradoxically, research that aims to contribute to the understanding of the correlation between ethnicity and health, can, by depicting ethnicity unproblematically as a potential SDH, lead to practices that themselves have a negative impact on health outcomes (24, 29).

### SDH and local realities

A third challenge is that even if we would succeed in catching the nature of a particular determinant such as ethnicity, gender, etc. in a stable and clear definition, and even if we would then be able to establish the exact nature of the relationship between that determinant and health, it would still be very hard to translate complex SDH models such as presented in the previous section in order to make them applicable to particular real life settings. As we showed in the previous section, there is general consensus [and even considerable evidence (1, 13, 14)] that inequities in health do arise as the outcome of complex (dynamic and interconnecting) pathways. However, even the most sophisticated and comprehensive models picturing these complexities generally do not offer more than suggestions of possible elements or pathways. Being designed as generic theoretical frameworks SDH models are bound to a certain level of abstractness and detachment. They may suggest *that* health outcomes and disparities are the products of complex processes, they may point out what kind of elements *may* be involved in those processes and they may stress that those elements *may* interconnect in different and dynamic ways and can result in an unlimited number of pathways to ill health, but they are not designed to explain in concrete detail what elements form what pathways in a *particular* situation/location. Nor can they describe how the health outcomes or disparities in a *particular* situation or local context come about. SDH models are necessarily abstract in the sense that they aim to provide a theoretical and not a factual overview of *potential* elements involved in *potential* pathways to ill health (disparities). How and what pathways to ill health (disparities) evolve depends, however, on how historical processes and international relationships work out in or mix with local contexts (19) and therefore differ from location to location, with each location or situation connecting the local with the global in its own particular way (6, 30). The following two examples based on extensive ethnographic fieldwork in Dominica and Colombia illustrate how historical processes and current international connections interact(ed) with local elements, leading to unique constellations of elements constituting pathways that determine Dominican and Colombian citizens’ health as well access to health services.

In her study into mother and child care in Dominica, Krumeich shows how child feeding practices of Dominican mothers are embedded in the women’s social-economical position, the cultural constructions of gender relations and the cultural conceptualization of body, health and illness, creating a specific role for ‘imported’ instant formula and for public health messages in this context (31, 32). In Dominica women generally speaking head their own household with their children. Their status is closely linked to the number of children and their ability to permanently hold on to a partner. Men’s status strongly depends on their virility, the number of partners they can conquer and the number of children they are able to procreate. Providing instant formula to their children is a way for fathers to prove their fatherhood. For women the formula is a welcome contribution to the household as it can be used for other children as well. Situations in which households parents live in different are rather common in the Caribbean and believed to have its origins in times of slavery when British Colonial Law didn’t provide a legal status for marriage and family among slaves and when traditional family structure couldn’t exist as slaves were often not able to live together as a family (33). In the specific development of gender relations, instant formula has become a way to regulate those relations. Furthermore, the use of instant formula is enhanced by the way gender relations and notions of the healthy body interact. Complex notions of bodily anatomy and the consequences for health and illness result in the idea that experiencing negative emotions, as for instance during conflicts with partners, disturb the balance in the body and spoils the mother’s milk. Additionally, it is believed that intercourse has a negative effect on the quality of breast milk too, while refraining from intercourse increases the risk of conflicts and negative emotions. So, instant formula is often seen as a better solution because women often experience situations that undermine the quality of breast milk. Finally, mothers consider the nurses who are mostly young and without children, as inexperienced and naïve and therefore don’t take the health education message on breast feeding very seriously. Without being able to describe those relations in the Dominican setting in detail, this example shows how complex interactions between several SDH such as local social and economic position, gender, cultural values and historical processes are highly context specific and influence the risk of ill health (not following the ‘breast is best’ recommendations of the WHO), attitudes towards health care professions and use of health care facilities.

In her ethnography on HIV/AIDS in Cartagena, Colombia, Quevedo-Gomez (34, 35) analyzes how complex relations between SDH contribute to increased vulnerability for HIV/AIDS for certain groups, especially married women. She shows how traditional gender roles rooted in ‘machismo’ create a specific understanding of the risk groups that are mentioned in public health messages. The integration of local structural culture of ‘machismo’ and the Public Health concept of risk groups leads to distinction between two types of AIDS carriers: street women and men who allow penetration by other men. According to machismo norms, that prescribe sexual modesty for women and give men the responsibility to protect their families on the one hand but are expected to have frequent sexual encounters the other hand, men are allowed to have occasional encounters with other men if their sexual appetite is still unsatisfied, as long as they penetrate. Homosexuality is highly taboo, but as long as men do not allow penetration by others they are not considered to be homosexual and do not consider themselves as being at risk. Women limit their risk of HIV through selection of the right partner, a man that can be relied on to comply with his responsibility to support and protect his family, including using a condom with other women and taking the penetrator role with other men. Furthermore Quevedo-Gomez illustrates how historically rooted racial hierarchy and economic dependency increase the vulnerability of married women. As work options, especially for the ‘blacks’, are really limited, black men are often forced to be active in sexual tourism, in order to be able to generate a family income. Machismo norms and economic dependency mean that women accept that their partner exchanges sex for basic goods with tourists, who often have a preference for individuals with a ‘black skin’. In this example SDH as international sex tourism, machismo norms, historically rooted racial inequalities and economic dependency interact in a complex but specific way, leading to a specific understanding of public health concepts and influencing their effectiveness.

While most SDH models are theoretical models and consequently remain abstract, policy and action usually involve concrete problems in particular, unique settings as presented in the examples. And it is these unique settings for which national and local governmental bodies, or organizations are responsible. They are dealing with real people in specific contexts. The two examples above show that effective policy and action can only be grounded in careful and comprehensive identification of relevant elements and in depth understanding of interconnections both at local as well as at national and international levels. If a picture that describes in detail what elements are combined in what ways in particular situations is lacking, there will be no concrete starting points for actual policy making and action.

### Universal guidelines in local contexts

A final challenge involves the introduction of policies, programs and other interventions into these unique constellations. As the aforementioned examples of Dominica and Colombia show, the public health messages to improve maternal and child health or reduce HIV/AIDS infections respectively do not result in the changes they aim to achieve. As shown those messages become part of complex local dynamics, where they have to counter locally embedded practices that are related to the social economic and culturally shaped realities people live in. Those examples don’t stand alone, but are part of a larger body of ethnographic studies that show unintended consequences of an unproblematic introduction of global standards in local context (36–39). Those studies show how innovations (technological, policy, educational, health promotion, etc.) themselves interconnect with the setting into which they are introduced. Not only impacting those settings, but in fact acting as SDH themselves. Based on theoretical insights from science and technology studies (40–43) those studies show that technologies, knowledge, professional routines, standards and practice, are ‘actors’ in their own right, with inherent logics about how reality works and in-build norms and values regarding how reality should work. As, due to globalization, these innovations are likely to have been developed or designed in and for other settings, logics about how reality works or normative notions regarding how reality should be made to work may also differ from the setting into which they are introduced.

## Discussion; lessons to be drawn from these challenges

The rise of the SDH discourse on the basis of statistical evidence that correlates ill health to SDH and attempts to picture causal pathways in comprehensive theoretical models led to widespread awareness that health and health disparities are the outcome of complex pathways of interconnecting SDH. Combining local and global dynamics, these pathways involve the social production of health and illness and have the potential to explain health inequities within or between populations or groups. Awareness of potential pathways however does not provide sufficient ground for policy and action. As we argued above, pathways are dynamic constellations resulting from the way in which historical and international relationships shape local circumstances. International, national and local governmental institutions cannot ground policy, action and intervention in mere awareness of the fact that pathways may exist, but should, as our examples suggest, look for detailed and in-depth analysis and understanding of the pathways constituting the ill health in the setting for which they are responsible. While the example on ethnicity shows this understanding should involve careful identification and contemplation of the different elements or SDH involved, the examples from Dominica and Colombia illustrate that in-depth insight the way these SDH interconnect to construct pathways to ill health is crucial for effective and sustainable policy and programming. Furthermore they make clear that the introduction of innovations can and often does bring along its own impact on health and health inequities. Being the products of a context in which history and international connections have created their own views of reality, its problems and suitable solutions, these ‘alien’ bodies may impact existing constellations, bringing forth entirely new pathways to health inequity.

Considering all this, we cannot but agree that the awareness that complex, intertwined SDH pathways underlie distribution of disease and risk of disease is of major importance. We also want to stress, however, that policies and actions that are overlooking the situatedness and uniqueness of particular local pathways, may not only undermine the development of effective and sustainable action, but could even transform into a SDH itself.

For effective policy making and programming insight into pathways to ill health and health inequity is crucial, but only if it is based on detailed, concrete analysis and only if it takes the unique particularities of each setting into consideration. Any policy intending to tackle illness and health inequities should therefore be preceded by extensive in-depth case studies. Against contemporary tendencies that require quick and visible results, invoked by the current call for (statistical) evidence and the need to come up with quick result to show a sponsor he gets value for money, we propose an approach that takes sufficient time to prepare policy and programming. To ensure reliability and comprehensiveness of the analysis all actors constituting the setting should be involved in such an analysis. Moreover, even after extensive preparation, ongoing monitoring of the effects, intended and unintended, remains important. In addition to an extensive preparation ongoing process evaluation involving all actors involved to flush out undesired impact for these actors is essential.

## References

[1] WHO (2010). A conceptual framework for action on the social determinants of health. Social determinants of health discussion paper 2.

[2] Solar O, Irwin A (2007). Towards a conceptual framework for the analysis and action on the social determinants of health.

[3] Labonté R, Laverack G (2008). Health promotion in action; from local to global empowerment.

[4] Labonté R (2012). Commentary: global action on social determinants of health. J Public Health Policy.

[5] Friel S, Marmot MG (2011). Action on the social determinants of health and health inequities goes global. Annu Rev Public Health.

[6] Birn AE, Pillay Y, Holtz TH (2009). Textbook of international health; global health in a dynamic world..

[7] Huynen MM, Martens P, Hilderink HB (2005). The health impacts of globalization: a conceptual framework. Global Health.

[8] 
Labonté R, Mohindra K, Schrecker T (2011). The growing impact of globalization for health and public health practice. Annu Rev Public Health.

[9] Lalonde M (1974). A new perspective on the health of the Canadians.

[10] Tones K, Green J (2004). Health promotion: planning and strategies.

[11] Bartholomew LK, Parcel GS, Kok G, Gottlieb NH (2006). Planning health promotion programs: an intervention mapping approach..

[12] Green LW, Kreuter M (2005). Health promotion planning: an educational and ecological approach..

[13] WHO (2010). Social determinants of health discussion paper series. http://www.who.int/social_determinants/publications/en/.

[14] Bonnefoy J, Morgan A, Kelly MP, Butt J, Bergman V (2007). Constructing the evidence base on the social determinants of health: a guide.

[15] National Collaborating Centre for Determinants of Health (2012). Assessing the impact and effectiveness of intersectoral action on the social determinants of health and health equity; an expedited systematic review.

[16] Labonté R, Torgerson R (2005). Interrogation globalization, health and development: towards a comprehensive framework for research, policy and political action. Crit Publ Health.

[17] Dahlgren G, Whitehead M (1991). Policies and strategies to promote social equity in health.

[18] Barton H, Grant M (2006). A health map for the local human habitat. J R Soc Promot Health.

[19] Labonté R, R Parker, M Sommer (2011). From International to Global: framing health in the New Millennium. Routledge handbook on global public health.

[20] Ayo N (2012). Understanding health promotion in a neoliberal climate and the making of health conscious citizens. Crit Publ Health.

[21] Galvin R (2002). Disturbing notions of chronic illness and individual responsibility: towards a genealogy of morals. Health.

[22] Graham H (2000). Understanding health inequalities.

[23] Marmot MG, Wilkinson RG (1999). Social determinants of health.

[24] Aronowitz R (2008). Framing disease: an underappreciated mechanism for the social patterning of health. Soc Sci Med.

[25] Helberg-Proctor A, Krumeich A, Meershoek A, Horstman A From defining ethnicity to making ethnicity: an analysis of biomedical and public health research in the Netherlands.

[26] Proctor A, Krumeich A, Meershoek A (2011). Making a difference: the construction of ethnicity in HIV and SDI epidemiological research by the Dutch National Institute for Public Health and Environment. Soc Sci Med.

[27] Epstein S (2007). Inclusion: the politics of difference in medical research.

[28] Yanow D, Van der Haar M (2013). People out of place: allochthony and autochthony in the Netherlands’ identity discourse-metaphors and categories in action. J Int Relat Dev.

[29] Meershoek A, Krumeich A, Vos R (2011). The construction of ethnic differences in work incapacity risks: analysing ordering practices of occupational physicians in The Netherlands. Soc Sci Med.

[30] Kickbusch I, Gleicher D (2011). Governance for health in the 21st century.

[31] Krumeich A (1994). The blessings of motherhood; health pregnancy and child care in Dominica.

[32] Krumeich A, Weijts W, Reddy P, Meijer-Weitz A (2001). The benefits of anthropological approaches for health promotion research and practice. Health Educ Res.

[33] Perry TL (1996). Symposium: family values, race, feminism and public policy. Santa Clara L. Rev.

[34] Quevedo-Gómez MC, Krumeich A, Abadia-Barrero CE, Pastrana-Salcedo EM, van den Borne HW (2012). Machismo, public health and sexuality-related stigma in Cartagena. Cult Health Sex.

[35] Quevedo-Gómez MC (2012). Participatory ethnography of HIV/AIDS in Cartagena, Colombia.

[36] Rak K, Janes GR (2004). Reproductive health in post-transition Mongolia: global discourses and local realities. Perspect Glob Dev Tech.

[37] Hadley M, Blum LS, Mujaddid S, Parveen S, Nuremowla S, Haque ME, Ullah M (2007). Why Bangladeshi nurses avoid nursing: social and structural factors on hospital wards in Bangladesh. Soc Sci Med.

[38] Hadley MB, Tuba M (2011). Local problems; local solutions: an innovative approach to investigating and addressing causes of maternal deaths in Zambia’s Copperbelt. Reprod Health.

[39] Livingston J, Cole J, Durham D (2007, pp. 164–89). Maintaining local dependencies; elderly women and global rehabilitation agendas in Southern Botswana. Generations and globalization.

[40] Bijker WE, Hughes TP, Pinch TJ (2012). The social construction of technological systems: new directions in the sociology and history of technology.

[41] Bijker WE (2009). Globalization and vulnerability; challenges and opportunities for SHOT around its fiftieth anniversary. Tech Cult.

[42] Timmermans S, Berg M (2003). The gold standard; the challenges of evidence-based medicine and standardization in health care.

[43] Engel N (2011). Local adaptations versus standardisation? Treatment delivery for multidrug resistant tuberculosis in India. Medische Antropologie.

